# Phenotypic rescue of a *Drosophila* model of mitochondrial ANT1 disease

**DOI:** 10.1242/dmm.016527

**Published:** 2014-05-08

**Authors:** Suvi Vartiainen, Shanjun Chen, Jack George, Tea Tuomela, Kaisa R. Luoto, Kevin M. C. O’Dell, Howard T. Jacobs

**Affiliations:** 1 BioMediTech and Tampere University Hospital, FI-33014 University of Tampere, Finland.; 2 Institute of Molecular, Cell and Systems Biology, University of Glasgow, Glasgow G12 8QQ, Scotland, UK.; 3 Research Program of Molecular Neurology, FI-00014 University of Helsinki, Finland.

**Keywords:** Adenine nucleotide translocase, Mitochondrial disease, Mitochondrial biogenesis, Alternative oxidase

## Abstract

A point mutation in the *Drosophila* gene that codes for the major adult isoform of adenine nuclear translocase (ANT) represents a model for human diseases that are associated with ANT insufficiency [*stress-sensitive B^1^* (*sesB^1^*)]. We characterized the organismal, bioenergetic and molecular phenotype of *sesB^1^* flies then tested strategies to compensate the mutant phenotype. In addition to developmental delay and mechanical-stress-induced seizures, *sesB^1^* flies have an impaired response to sound, defective male courtship, female sterility and curtailed lifespan. These phenotypes, excluding the latter two, are shared with the mitoribosomal protein S12 mutant, *tko^25t^*. Mitochondria from *sesB^1^* adults showed a decreased respiratory control ratio and downregulation of cytochrome oxidase. *sesB^1^* adults exhibited ATP depletion, lactate accumulation and changes in gene expression that were consistent with a metabolic shift towards glycolysis, characterized by activation of lactate dehydrogenase and anaplerotic pathways. Females also showed downregulation of many genes that are required for oogenesis, and their eggs, although fertilized, failed to develop to the larval stages. The *sesB^1^* phenotypes of developmental delay and mechanical-stress-induced seizures were alleviated by an altered mitochondrial DNA background. Female sterility was substantially rescued by somatic expression of alternative oxidase (AOX) from the sea squirt *Ciona intestinalis*, whereas AOX did not alleviate developmental delay. Our findings illustrate the potential of different therapeutic strategies for ANT-linked diseases, based on alleviating metabolic stress.

## INTRODUCTION

*Drosophila melanogaster* is a convenient animal model for human genetic diseases, including those that are associated with mitochondrial dysfunction. Autosomal dominant progressive external ophthalmoplegia (adPEO) is a relatively common presentation of mitochondrial disease and is associated with multiple mitochondrial (mt)DNA deletions in muscle and other tissues (Suomalainen and Kaukonen, 2001). It is caused, typically, by single gene mutations in nuclear genes that are directly, or indirectly, involved in mtDNA maintenance – including those encoding the two subunits of DNA polymerase γ, POLG1 (Van Goethem et al., 2001) and POLG2 (Longley et al., 2006), the mitochondrial helicase Twinkle (C10orf2) (Spelbrink et al., 2001), the ribonucleotide reductase small subunit RRM2B (Tyynismaa et al., 2009) and the muscle-specific isoform of the adenine nucleotide translocase ANT1/SLC25A4 (Kaukonen et al., 2000). adPEO-causing mutations have been mapped to different regions of the *ANT1* coding sequence (Kaukonen et al., 2000; Napoli et al., 2001; Komaki et al., 2002; Deschauer et al., 2005). Functional studies of two such mutants – A114P and V289M – in cultured myotubes revealed that, for a given mitochondrial inner membrane potential, the rate of adenine nucleotide exchange was substantially decreased, and, under some conditions, the reversed transport of ATP into mitochondria was predicted to occur readily (Kawamata et al., 2011). Earlier modeling of these mutants in yeast (Fontanesi et al., 2004) also concluded that they impair translocase activity and lead to a defect in oxidative phosphorylation (OXPHOS). *ANT1* encodes the major isoform of the translocase that is found in post-mitotic tissues – such as the heart, skeletal muscle and brain – whereas three other isogenes encode isoforms of the translocase in other cells and tissues (Chevrollier et al., 2011).

AdPEO is a late-onset disorder, but *ANT1* mutations are also associated with more severe early-onset diseases that show recessive inheritance. These include mitochondrial myopathy and cardiomyopathy due to complete, or almost complete, loss of translocase function (Palmieri et al., 2005), which also manifests in mice in which *Ant1* has been knocked out (Graham et al., 1997; Narula et al., 2011). ANT1 deficiency has also been proposed to be a major contributor to the clinical features of Sengers syndrome, which comprises congenital cataract, hypertrophic cardiomyopathy and skeletal myopathy (Jordens et al., 2002). In this case, the effects on ANT1 are secondary because the primary mutation is in *AGK*, encoding acylglycerol kinase (Mayr et al., 2012), which results in mitochondrial phospholipid defects. Dysregulation of ANT1 has also been suggested to be involved in the pathogenesis of inclusion-body myositis (Barca et al., 2013) and Rett syndrome (Forlani et al., 2010).

The molecular pathology of ANT1-associated disease has been extensively studied using samples taken from affected individuals, as well as using the *Ant1*-knockout mouse. However, these studies have certain limitations; in human cells, the ability to recapitulate the exact *in vivo* physiological conditions in affected tissues – for example, the failing heart – is severely limited. As indicated above, *Ant1*-knockout mice exhibit dilated cardiomyopathy that is associated with a progressive decline of cardiac functions, ending in chronic heart failure (Narula et al., 2011). Conversely, *Ant1* overexpression is cardioprotective (Wang et al., 2009). The knockout exhibits lactic acidosis and the hyperproliferation of abnormal mitochondria in muscle (Graham et al., 1997). The increased production of reactive oxygen species (ROS) in mitochondria that have been isolated from the affected tissues of knockout animals is also associated with mitochondrial genomic damage (Esposito et al., 1999). More surprisingly, loss of *Ant1* in the brain entrains increased mitochondrial membrane potential and resistance to apoptosis, leading to the suggestion that the protein confers natural susceptibility to excitotoxicity (Lee et al., 2009). Muscle mitochondria from the knockout mouse show decreased proton leak (Brand et al., 2005), which might explain these observations, but does not necessarily elucidate the functional consequences of pathological mutations.

**TRANSLATIONAL IMPACT**

**Clinical issue**

A variety of genetic mutations have been implicated in mitochondrial myopathies, which are characterized by muscular weakness and neurological abnormalities. One cause of mitochondrial myopathy is mutation of the gene that codes for the adenine nucleotide translocase (ANT). ANT is the transporter protein that shuttles ADP and ATP across the inner mitochondrial membrane, enabling the energy released from catabolism to be made available to the rest of the cell. ANT deficiency could be a common manifestation of mitochondrial disease, because it can also be produced by other abnormalities affecting the organelle; for example, defects in the mitochondrial membrane phospholipid composition. In this study, the authors used *Drosophila* to explore the effects of ANT deficiency and test possible strategies to overcome the defect.

**Results**

The authors characterized the phenotype of *sesB^1^* mutant flies, which carry a mutation in the major ANT-encoding gene of *Drosophila*. Mutant flies were profiled at the organismal (developmental, behavioral), metabolic and molecular genetic levels. *sesB^1^* flies showed developmental retardation, epileptic seizures upon mechanical stress, hearing impairment, female sterility, defective male courtship and greatly decreased lifespan. There was an accumulation of lactate, an end-product of glucose breakdown that is potentially toxic at high concentrations. The expression of many genes for metabolic functions was altered, and, in females, many genes involved in egg production were downregulated. The authors showed that developmental delay and seizures could be partially alleviated by transferring the *sesB^1^* mutation to a mitochondrial DNA background that is thought to increase mitochondrial biogenesis. A minor effect on the sensitivity of mutant flies to mechanical-stress-induced seizures was produced by increased expression of the fly homolog of *PGC1-α*, the proposed ‘master regulator’ of mitochondrial biogenesis. Female sterility, but not the other phenotypes, was substantially rescued by expression of the alternative oxidase (AOX) from the sea squirt *Ciona intestinalis*, which has previously been shown to mitigate oxidative stress caused by mitochondrial inhibition.

**Implications and future directions**

The authors’ comprehensive analysis of *sesB^1^* mutant flies demonstrates that ANT deficiency causes a host of developmental and neurological phenotypes that are underpinned by abnormalities in metabolism. They provide evidence that pharmacological strategies aimed at boosting mitochondrial biogenesis, as well as genetic therapies based on AOX, could be effective treatments for mitochondrial myopathies and other human diseases that are associated with ANT deficiency. A useful next step will be to analyze in more detail the mechanisms by which these interventions alleviate the disease-like features of *sesB^1^* flies, with the aim of eventually translating these findings to human therapies.

The *Ant1*-knockout mouse represents, by definition, complete loss of function, which does not appear to be the case for human ANT1 in disease, except in rare cases (Palmieri et al., 2005). However, the fact that ANT is encoded in mammals by a multigene family means that the knockout of one isoform might entrain effects on the regulation of others, which can also compensate for any defect. This has been hypothesized to account for the lack of retinal pathology in the *Ant1*-knockout mouse (Phillips et al., 2010). For these reasons, a more appropriate model is required that is based on actual disease-relevant mutations and in which the effects of genetic redundancy can be set aside.

In *Drosophila*, ANT is also encoded by a small gene family. In this case, however, the major isoform, which is encoded by the gene *stress-sensitive B* (*sesB*; FlyBase annotation CG16944), is ubiquitously expressed at a high level, whereas the mRNA for the second isogene, *Ant2* (FlyBase annotation CG1683), is found only at low levels, except in testis (www.flyatlas.org). A mutant allele of *sesB*, *sesB^1^*, exhibits a phenotype of susceptibility to mechanical-stress-induced seizures (‘bang sensitivity’) and developmental delay (Zhang et al., 1999), as well as physiological abnormalities in the tubule (Terhzaz et al., 2010). The combination of developmental delay and bang sensitivity is shared with other *Drosophila* mutants that exhibit perturbed mitochondrial OXPHOS, including alleles of *knockdown* (*kdn*), which encodes citrate synthase (Fergestad et al., 2006), and *technical knockout* (*tko^25t^*), which encodes mitoribosomal protein S12 (Royden et al., 1987; Shah et al., 1997; Toivonen et al., 2001). A further bang-sensitive mutant in the gene *easily shocked*, which encodes ethanolamine kinase (Pavlidis et al., 1994), shares the phenotype of decreased steady-state ATP levels with *sesB^1^*, *tko^25t^* and some alleles of *kdn* (Fergestad et al., 2006) and might also act mitochondrially. *tko^25t^* manifests multiple deficiencies of the respiratory chain and OXPHOS, and exhibits an impaired response to sound and defective male courtship (Toivonen et al., 2001).

**Fig. 1.**
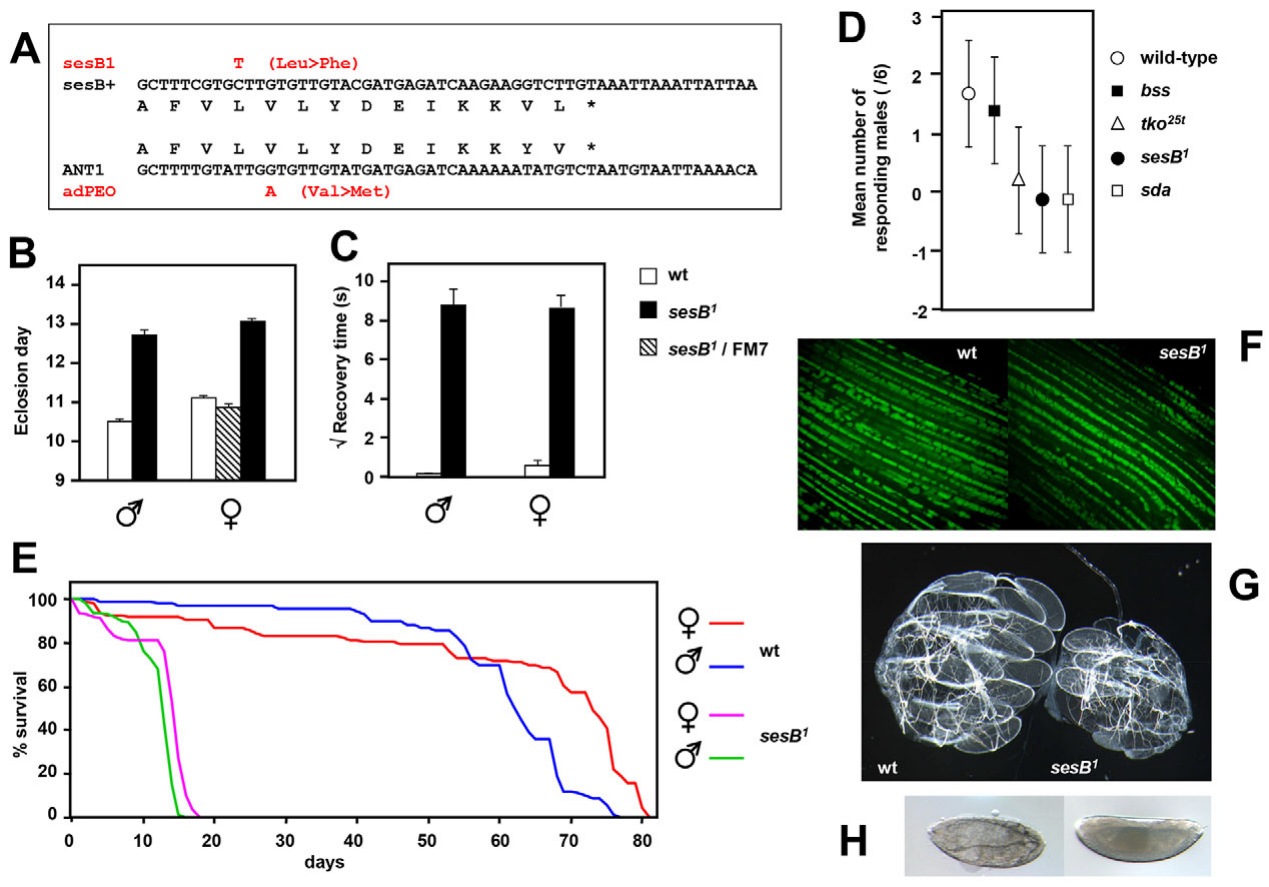
**Genotype and phenotype of *sesB^1^* flies.** (A) Aligned partial sequences of *sesB* mRNA, isoform A (sesB+), commencing at nucleotide 962 of NCBI database entry NM_078554, and human *ANT1* mRNA (NCBI database entry NM_001151 from nucleotide 863). The corresponding amino acid sequences and the base substitutions (red) and corresponding amino acid changes in the *sesB*^1^ allele, verified in the strain analyzed here, are also shown. The *ANT1* gene mutation shown here is that reported in an Italian family with adPEO (Kaukonen et al., 2000). Each mutation results in the substitution of a hydrophobic residue in transmembrane segment VI of the protein (Pebray-Peyroula et al., 2003) by another hydrophobic residue. (B) The day of eclosion of flies of the genotypes indicated; means±s.e.m. are shown from five replicate vials. FM7 is the X-chromosomal balancer chromosome used to maintain heterozygosity for *sesB^1^*. (C) The square-root of the time taken to recover from mechanical shock (‘bang sensitivity’) for batches of ≥25 flies of each sex and genotype that were individually analyzed; means±s.e.m. are shown. The day of eclosion and bang sensitivity of *sesB^1^* flies were significantly different from those of wild-type (wt) or heterozygous flies of the same sex (*P*<0.01). (D) Response of adult males of the indicated strains to sound. The mean number of responders±s.d. amongst groups of six males from three replicate groups of each genotype are shown. Bang-sensitive strains are: *tko^25t^*, point mutant of *technical knockout*, which encodes mitoribosomal protein S12 (Shah et al., 1997); *bss*, *bang senseless*, a point mutant of *paralytic*, which encodes a voltage-gated Na^+^ channel (Parker et al., 2011); *sesB^1^*; and *sda*, *slamdance*, which encodes aminopeptidase N (Zhang et al., 2002). *tko^25t^*, *sesB^1^* and *sda* are non-responsive, whereas *bss* has normal hearing. (E) Lifespan curves for *sesB^1^* and wild-type control flies of the sexes indicated (groups of 100 flies of each sex and genotype) when maintained at 25°C. Three replicate experiments gave similar results – i.e. mean lifespans of 11–13 days for *sesB^1^* males and 13–18 days for *sesB^1^* females. (F) GFP that was targeted to the mitochondria was expressed in indirect flight muscles of wild-type and *sesB^1^* flies (in both genotypes in the presence of a transgene encoding UAS-mito-HA-GFP and the *Mef2-GAL4* driver). The mitochondrial network appears normal in *sesB^1^*. (G) Dark-field micrograph of the dissected ovaries from 2-day-old wild-type and *sesB^1^* females. (H) Phase-contrast micrograph of developing embryos from eggs laid by *sesB^1^* females. The vast majority of embryos failed to hatch, and only a tiny number (~0.1%) completed development.

Importantly, when the human and *Drosophila* sequences are aligned, point mutation L289F in *sesB^1^*, a hydrophobic substitution in the final transmembrane-spanning helix of the protein (Pebay-Peyroula et al., 2003), is adjacent to the site of the hydrophobic substitution V298M in adPEO (Fig. 1A). Although the *sesB^1^* phenotype differs in its tissue specificity from that of human ANT1-associated diseases, it presents a more disease-relevant genotype than the *Ant1*-knockout mouse, and is effectively free of the effects that are attributable to genetic redundancy. It therefore offers a convenient tool for understanding the metabolic and cellular consequences of ANT1 deficiency, and for testing strategies to compensate for pathological defects.

In previous studies, we used transcriptomic analysis to profile changes in gene expression in the *tko^25t^* mutant compared with wild-type flies. The results implied that there is a metabolic shift in *tko^25t^* flies, with diversion of pyruvate to lactate to regenerate NAD^+^, increased mobilization of dietary fat and protein, and the induction of anaplerotic pathways to maintain the flow of carbon skeletons for biosynthesis (Fernández-Ayala et al., 2010). We have also shown that the *tko^25t^* phenotype can be partially suppressed by additional dosage of the mutant gene (Kemppainen et al., 2009) or by altering mtDNA background (Chen et al., 2012), which appeared to act by increasing mitochondrial biogenesis. Therefore, we aimed to analyze metabolism and gene expression in *sesB^1^* flies and to test different strategies to compensate the mutant phenotype, using similar approaches as those used previously for *tko^25t^*.

Because the *sesB^1^* and *tko^25t^* phenotypes overlap, transcriptomic analysis can address what underlies the differences and similarities between the two mutants at the molecular level. These two classes of mutant – i.e. the one affecting mitochondrial protein synthesis, the other affecting intracellular nucleotide transport and synthesis – are common causes of mitochondrial disease (Saada, 2004; Sharer, 2005; Jacobs and Turnbull, 2005). Therefore, understanding both the common and the specific features of their molecular pathology should lead to more accurate diagnosis and targeted therapy.

In the mouse, transcriptomic studies have, thus far, been reported only in the skeletal muscle of 9- to 12-month-old animals for a limited set of known mitochondrially targeted gene products (Subramaniam et al., 2008), providing evidence of a paradoxical or futile upregulation of mitochondrial biogenesis. Here, we extend this to a more comprehensive analysis involving the whole genome and the whole organism in the *Drosophila melanogaster* model.

## RESULTS

### Strain verification and phenotyping

In order to analyze metabolism and gene expression in *sesB^1^* mutant flies, and to test possible strategies for suppressing the mutant phenotype, we first verified the genotype (Fig. 1A) and phenotype (Fig. 1B–H) of the available strain, which was further backcrossed into standard wild-type backgrounds (Oregon R and Canton S). Compared with *tko^25t^* (Chen et al., 2012; Kemppainen et al., 2009), the developmental delay of *sesB^1^* was less pronounced, notably in males (Fig. 1B), whereas the bang sensitivity of *sesB^1^* adults, when tested at 3 days of age, was greater (Fig. 1C). *sesB^1^* flies were also deaf (Fig. 1D), as judged by a behavioral assay for response to sound, as used previously to study *tko^25t^* flies (Toivonen et al., 2001). Despite the short lifespan (Fig. 1E), the muscle mitochondrial network in young adult flies was intact (Fig. 1F), unlike that seen in the case of other degenerative mutations that affect mitochondrial functions, such as *Pink1^5^* (Deng et al., 2008). Female flies were essentially sterile. At 2 days of age, ovary size was, typically, decreased (Fig. 1G), although eggs were eventually produced in normal, or near normal, numbers. However, eggs that were laid developed only as far as the late-embryo stage (Fig. 1H), with a tiny number of survivors reaching adulthood (~0.1% of the number of progeny from control crosses). Males were fertile but, in courtship assays, they displayed a defect similar to that observed previously for *tko^25t^* (Toivonen et al., 2001): in two separate experiments, only one of 11 and one of 13 individual *sesB^1^* males reached copulation with single wild-type females, whereas ten out of ten wild-type males successfully copulated.

### *sesB^1^* flies have defects in respiration and OXPHOS

**Fig. 2.**
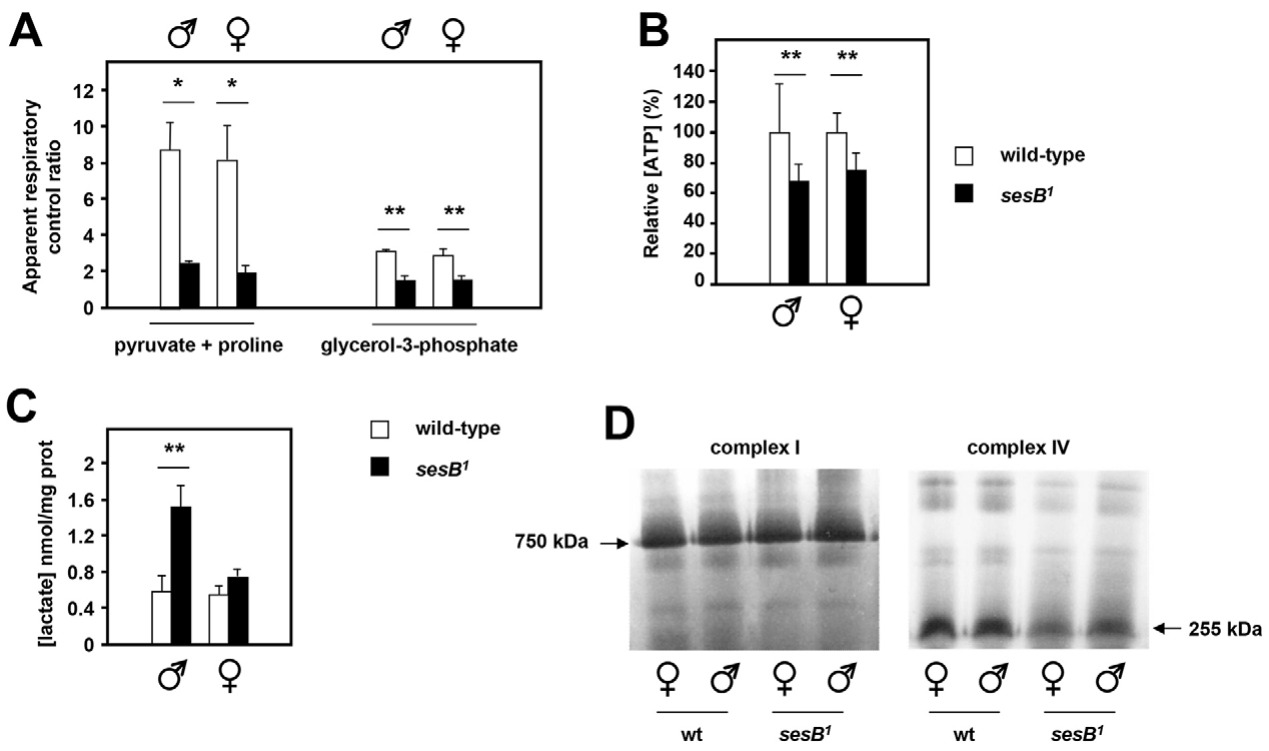
**Mitochondrial metabolism in *sesB^1^* flies.** (A) Apparent respiratory control ratio (RCR; the relative rate of oxygen consumption before and after the addition of ADP) of isolated mitochondria from flies of the strains and sexes indicated, using different substrate mixes, based on the data shown in Table 1. Note that the value obtained with the pyruvate and proline substrate mix is not a true RCR, because of the secondary activation of isocitrate dehydrogenase by ADP (Miwa et al., 2003). However, a similar trend is observed with both substrate mixes. (B) Steady-state ATP levels in the extracts from flies of the strains and sexes indicated, normalized to the value in wild-type (wt) flies in the given sex. The means of results from five or six biological replicates for each group are shown. (C) Steady-state lactate levels in extracts from 10-day-old flies of strains and sexes indicated. *n*=6 for males, *n*=3 for females. (A–C) Means±s.d., *P<0.05, **P<0.01 by using Student’s *t*-test. (D) Representative blue native electrophoresis gels, which were stained histochemically for complex I and IV, of extracts from flies of the strains and sexes indicated. Arrows indicate the major complexes that were stained; their apparent molecular weights were extrapolated from the migration of markers.

In order to test how the *sesB^1^* mutation affects the properties of the ANT protein, we measured the rate of respiration of isolated mitochondria from *sesB^1^* and control flies in the presence of different substrates, with and without the addition of 1 mM ADP. The respiratory control ratio (RCR), computed as the respiration rate in the presence of ADP divided by that in its absence, represents the degree to which respiration is limited by ATP synthesis, which is in turn dependent on ANT activity. On both complex-I- and complex-III-linked substrate mixes, mitochondria from *sesB^1^* flies showed a significant drop in the apparent RCR (Table 1; Fig. 2A), consistent with decreased ANT activity limiting both ATP synthesis and respiration. Note, however, that because of the secondary activation of isocitrate dehydrogenase by ADP, the complex-I-linked data is not a true reflection of the RCR (Miwa et al., 2003). There was a 50% decrease in RCR, even using the complex-III-linked substrate glycerol-3-phosphate.

**Table 1.**
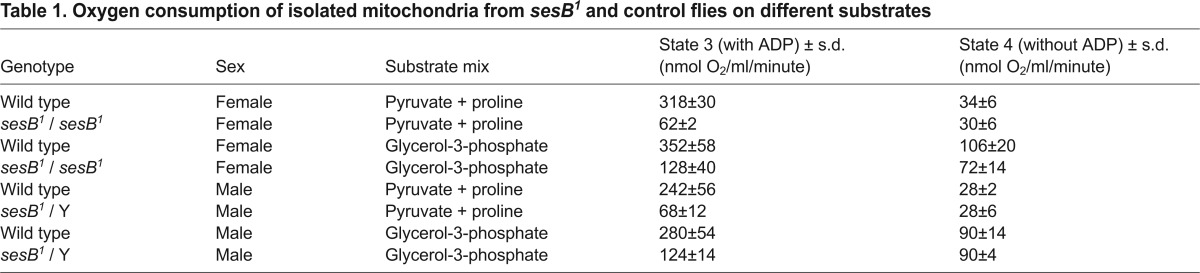
Oxygen consumption of isolated mitochondria from *sesB^1^* and control flies on different substrates

Steady-state ATP levels were also consistently lower in *sesB^1^* flies than control flies (Fig. 2B). Under conditions where respiration is limiting, re-oxidation of NADH should depend on cytosolic enzymes, such as lactate dehydrogenase. This was reflected here by increased levels of lactate (Fig. 2C). In order to investigate the effects of the *sesB^1^* mutation on the respiratory chain, we analyzed the activity of complex I and IV in mitochondrial extracts from *sesB^1^* and control flies, using blue-native electrophoresis (BNE) combined with histochemical staining (Fig. 2D; supplementary material Fig. S1). Complex I activity in *sesB^1^* was indistinguishable from that in wild-type flies, but there was a clear decrease in complex IV activity, especially in females.

### Changes in gene expression in *sesB^1^* are partly sex-specific

We implemented a gene expression analysis of *sesB^1^* flies using procedures identical to those used previously to analyze *tko^25t^*. To minimize strain-specific effects, the flies analyzed using the Affymetrix *Drosophila* 2.0 microarray were, as in the case of *tko^25t^*, an F1 hybrid of *sesB^1^* that had been backcrossed to Canton S or Oregon R backgrounds, and the results were compared with those of a corresponding control F1. The changes in the expression of a subset of genes were then verified by quantitative RT-PCR (Table 2). Analyses were conducted separately for males and females, in order to clarify the bisexual versus sex-specific aspects of the mutant phenotype.

**Table 2.**
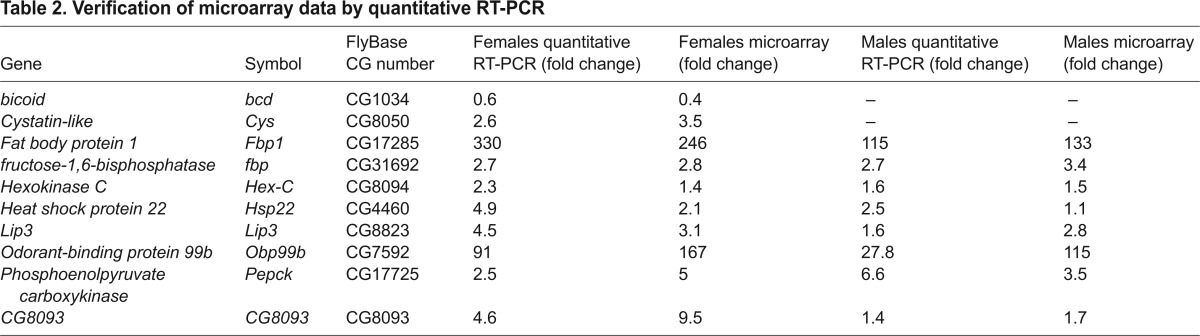
Verification of microarray data by quantitative RT-PCR

**Fig. 3.**
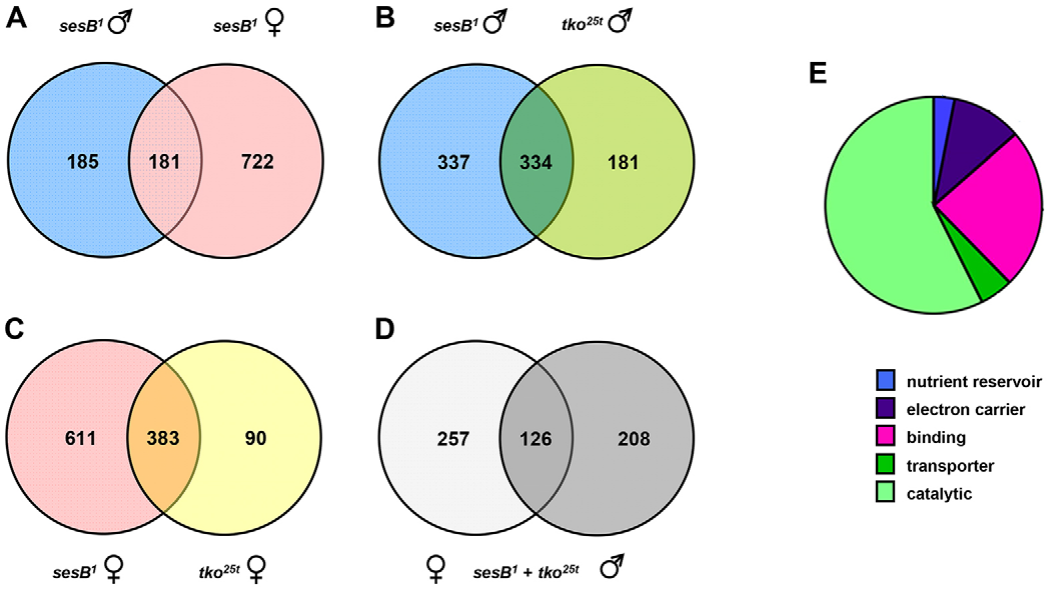
**Summary of transcriptomic analysis of *sesB^1^* flies.** (A–D) Diagrammatic representation of degree of overlap between probesets that had altered expression from wild type; fly strains and sexes are indicated. For details, see supplementary material Tables S1–S7. (E) GO classification by molecular function of the genes that showed altered expression in *sesB^1^* flies of both sexes (panel A; supplementary material Table S1).

After statistical filtering, we identified 903 probesets that detected genes whose expression in *sesB^1^* females differed at least twofold from that of wild-type controls (supplementary material Table S1), and 366 such probesets in males (supplementary material Table S2). Only 181 probesets were shared between the sexes (Fig. 3A; supplementary material Table S3), 177 of which showed directionally congruent changes, most of which were quantitatively very similar, with eight times as many genes that were upregulated as downregulated. Gene ontology (GO) analysis revealed that the genes represented by the 181 bisexually-regulated probesets were mostly annotated with molecular functions in metabolism (Fig. 3E). Of the probesets that were regulated in a sex-specific manner, downregulation was slightly more prevalent than upregulation. We inspected in more detail the expression patterns and GO terms of the 20 most up- or downregulated sex-specific annotated genes in each sex (Table 3), which revealed a consistent pattern. The sex-specific upregulated genes fell mostly into similar categories as those regulated bisexually, suggesting that these represent pathways regulated in a similar way in the two sexes, but in which specific genes were rejected in one sex. This might have been due to the stringent statistical filtering, or, alternatively, to the quantitative limitations of the method, in comparison with quantitative PCR (see Table 2). Some of the genes that were specifically downregulated in males were also in these categories. However, the remaining genes that were downregulated in males, and almost all of those that were downregulated in females, were associated with reproduction and broadly consistent with the organismal phenotype. Thus, the genes that were most downregulated in females were predominantly involved in oogenesis – including chorion formation and cell division functions – which is consistent with defective oocyte development. In males, the most prominent set of sex-specific downregulated genes were expressed primarily, or exclusively, in the accessory glands and have been implicated in pheromone perception and other aspects of courtship behavior, in which *sesB^1^* males were defective. Global GO analysis of the annotated genes that showed alterations in their expression in a sex-specific manner in *sesB^1^* flies (supplementary material Tables S4, S5) was consistent with this ‘top 20’ analysis, although many ovary-specific or ovary-enriched genes that are involved in oogenesis are listed under other categories – such as anatomical structure development, cell cycle, cell differentiation, cell division, chromosome or cytoskeleton organization, mitosis, chromosome segregation or DNA metabolic process.

**Table 3.**
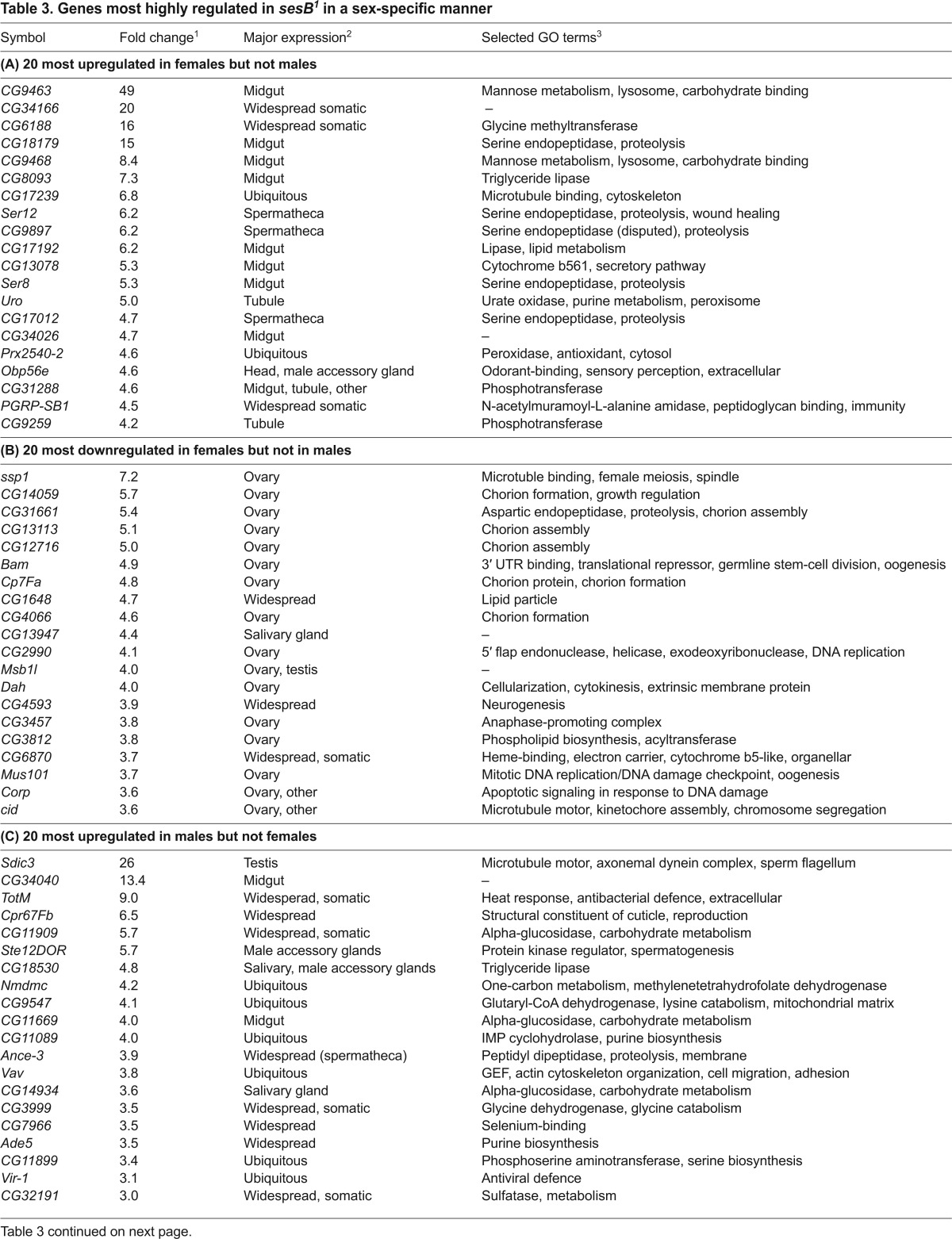

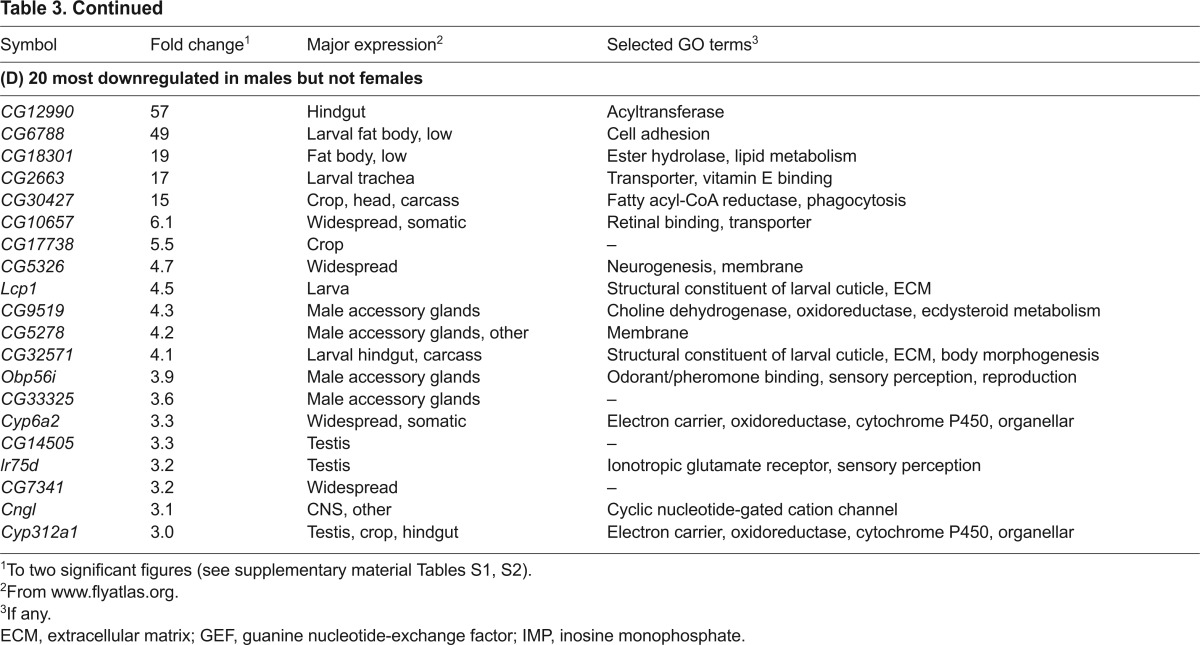
Genes most highly regulated in *sesB^1^* in a sex-specific manner

### Changes in gene expression in *sesB^1^* flies overlap those in *tko^25t^*

Next, we compared the patterns of change in gene expression in *sesB^1^* flies with those seen in *tko^25t^* (Fig. 3B–D; supplementary material Tables S6–S10) in order to reveal both common and distinct pathways that are affected in these models of two major classes of mitochondrial disease. For coherence with the previous analysis of *tko^25t^* (Fernández-Ayala et al., 2010), we applied a slightly more relaxed criterion of a statistically robust expression difference of ≥1.5-fold. In males, approximately two-thirds of the probesets that showed altered expression in *tko^25t^* were also altered in *sesB^1^*, and half of the genes that showed altered expression in *sesB^1^* were also altered in *tko^25t^* with, once again, all but a handful of genes showing the same directional difference in their expression, most of which were quantitatively very similar (Fig. 3B; supplementary material Table S7). In females, the vast majority of the probesets that showed altered expression in *tko^25t^* were also altered in *sesB^1^* flies (383 of 473 probesets), whereas a larger number of the genes that showed altered expression in *sesB^1^* females (611 probesets) did not appear in the *tko^25t^* list (Fig. 3C; supplementary material Table S6). Almost all of the genes that showed the greatest changes in expression in *tko^25t^* flies, whether regulated bisexually or in a sex-specific manner, were altered to a similar degree in *sesB^1^* (supplementary material Table S10). Of those showing the greatest sex-specific changes in *sesB^1^* females, as listed in Table 2, 15 out of 20 of those that were upregulated were also upregulated in *tko^25t^*, whereas only seven out of the 20 most downregulated genes in *sesB^1^* females were also downregulated in *tko^25t^* females. Considering both mutant backgrounds, 126 probesets were regulated in a similar manner in both sexes in both mutant strains (Fig. 3D; supplementary material Table S8), whereas a larger number were sex-specific (Fig. 3D; supplementary material Table S9). GO analysis of the genes that were up- or downregulated in *sesB^1^* females but not in *tko^25t^* females placed these genes in diverse categories. However, more than half of the 122 annotated genes that were downregulated (but almost none of the upregulated genes) are expressed uniquely, or predominantly, in the female reproductive system (www.flyatlas.org).

### Altered expression of metabolic genes in *sesB^1^* reflects the biochemical phenotype

The observed changes in the expression of metabolic genes in *sesB^1^* can be broadly rationalized, as in the case of *tko^25t^*, in the following manner. First, the bulk of ATP production is shifted to glycolysis, with conversion of pyruvate to lactate to regenerate NAD^+^. Second, fatty acids and amino acids are mobilized, and anaplerotic pathways induced to maintain the flow of carbon skeletons for biosynthesis. Thus, there was upregulation of a set of enzymes that converged on pyruvate and acetyl-CoA in both sexes – fructose-1,6-bisphosphatase, aldolase, phosphoenolpyruvate carboxykinase (Pepck), lactate dehydrogenase, acetyl-CoA synthetase and aldehyde dehydrogenase, as well as alcohol dehydrogenase in females (supplementary material Table S11). A second Pepck isogene (CG10924) and a testis-specific lactate dehydrogenase gene were downregulated (in males). There was also an overlap between the sets of genes that showed altered expression in *sesB^1^* and those that are altered under starvation (Farhadian et al., 2012), although some of the changes were in opposite directions (supplementary material Table S12).

### Testing for phenotypic complementation of *sesB^1^* by altered mtDNA background or *spargel* overexpression

In previous work, we have described the partial alleviation of the phenotypes of *Drosophila* mutants that represented mitochondrial disease models associated with cytochrome oxidase deficiency (Fernández-Ayala et al., 2009; Kemppainen et al., 2014), complex I deficiency (Sanz et al., 2010a), DNA polymerase γ disease (Humphrey et al., 2012) and disorders of mitochondrial protein synthesis (Kemppainen et al., 2009; Chen et al., 2012). The similar organismal phenotypes and the overlapping patterns of altered gene expression in *tko^25t^* and *sesB^1^* led us to question whether the same strategies might be effective in *sesB^1^* flies.

We have previously reported that the BER-1 mtDNA background, which produced a clear phenotypic improvement in *tko^25t^*, had no effect on the developmental phenotype of *sesB^1^*, although there was a partial rescue of its short lifespan (Chen et al., 2012). Note, however, that observations of eclosion timing and bang sensitivity that we reported in that publication were made after backcrossing *sesB^1^* directly into the BER-1 cytoplasmic background, which remained infected with the endosymbiont *Wolbachia*. Furthermore, the continued presence of *Wolbachia* compromised the increase in the lifespan of *sesB^1^* that was conferred by the BER-1 mtDNA background (see figure 3 in the supplementary data of Chen et al., 2012). Therefore, we investigated whether the BER-1 mtDNA background, in the absence of *Wolbachia*, was able to rescue the developmental phenotype of *sesB^1^*.

**Fig. 4.**
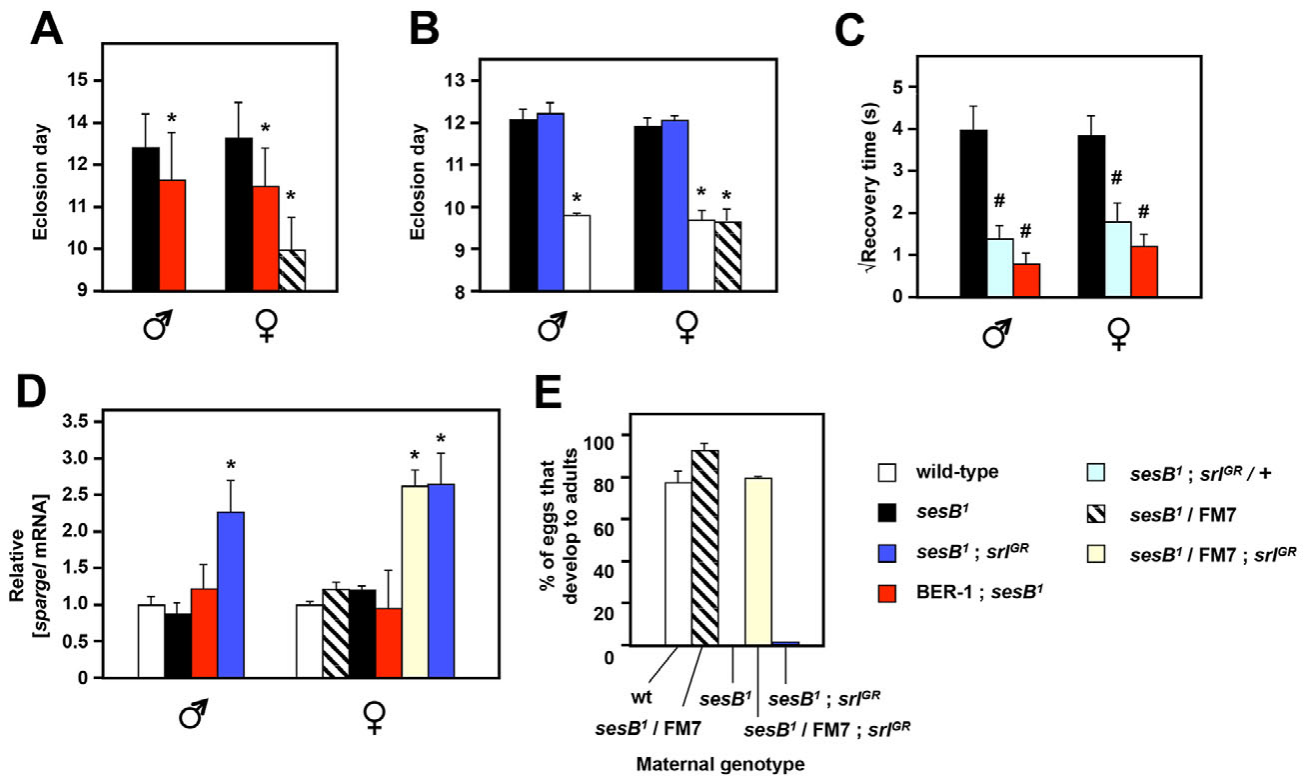
**Testing rescue of *sesB^1^* by altered mtDNA background or *spargel* overexpression.** (A,B) The day of eclosion of flies of the genotypes and sexes indicated is shown; means±s.d. from three replicate experiments. FM7 is the X-chromosomal balancer chromosome used to maintain heterozygosity for *sesB^1^*, which gives a wild-type phenotype. BER-1;*sesB^1^* denotes cybrid flies (BER-1 mtDNA, *sesB^1^* mutation and nuclear background). *srl^GR^* (an additional genomic copy of *spargel*) was homozygous, as indicated. (C) The square-root of the recovery time from mechanical shock (bang sensitivity) of flies of the genotypes and sexes indicated; means±s.e.m. are shown. *srl^GR^* flies in this experiment were hemizygous. The number of individually analyzed flies of each genotype was between 19 and 24 for the different classes. In B and C, ^#^*P*<0.05, *P<0.01 between *sesB^1^* and other data classes. Note that *t*-test or ANOVA cannot be meaningfully implemented for A owing to the skewed distribution of the day of eclosion in the BER-1 mtDNA background; hence the data are only indicative. The partial rescue of bang sensitivity by overexpression of *spargel* is statistically significant, but because an entire chromosome is replaced in this experiment, we cannot exclude a contribution of the genetic background. (D) Relative *spargel* mRNA levels, in flies of the sex and genotype indicated, normalized to the value in the same sex for wild-type flies. Only the lines that overexpressed *spargel* showed a significant increase in the mRNA, denoted by *, as above. (E) The mean proportion of eggs that developed into adults for the offspring of females, of the genotypes indicated, mated to wild-type males. Means±s.d. from three replicate experiments are shown.

BER-1 females, after removal of *Wolbachia* by tetracycline treatment (Dobson et al., 2002; Chen et al., 2012), were backcrossed for more than five generations to *sesB^1^* in order to create a cybrid strain that contained BER-1 mtDNA but the original nuclear genome of *sesB^1^*. Phenotypic testing of the cybrid flies indicated a partial rescue of both developmental delay (Fig. 4A) and bang sensitivity (Fig. 4C). In previous work, we found that the partial rescue of *tko^25t^* by the BER-1 mtDNA background was accompanied by an increased expression level of the *Drosophila PGC-1α* homolog *spargel* (Chen et al., 2012) and, furthermore, that overexpression of *spargel* was able to phenocopy the effect. However, in the *sesB^1^* flies we did not find any effect of the BER-1 mtDNA background on the expression level of *spargel* (Fig. 4D), indicating that this is not the mechanism of rescue.

We also tested directly whether *spargel* overexpression was able to suppress the *sesB^1^* phenotype. Despite an apparent partial rescue of bang sensitivity (Fig. 4C), there was no rescue of developmental delay (Fig. 4B), despite the increase in *spargel* expression at the RNA level that was conferred by the extra copy of the gene (Fig. 4D). *spargel* overexpression produced only a minimal rescue of the sterility of *sesB^1^* females when mated to wild-type males (Fig. 4D), which was also the case for the BER-1 flies, with only a slight increase in the number of survivors as compared with that resulting from control crosses.

### Phenotypic complementation of *sesB^1^* by AOX expression

Use of a respiratory chain bypass for complexes III and IV, via the alternative oxidase (AOX) from the sea squirt *Ciona intestinalis*, should facilitate electron flow through complex I, if the latter is inhibited by a limitation on ATP synthesis imposed by ANT dysfunction. This is predictable from the fact that complex I, combined with AOX, pumps fewer protons per electron than the regular respiratory chain. Any additional restriction due to a decrease in complex IV activity (Fig. 2D) might also be circumvented. Furthermore, respiration through complex II or other dehydrogenases that donate electrons to ubiquinone should also be facilitated under such conditions, because, when combined with AOX, they do not pump any protons. Thus, AOX expression should relieve *sesB^1^* phenotypes that result from disturbed redox or metabolic homeostasis, but not those caused by decreased ATP synthesis per se.

**Fig. 5.**
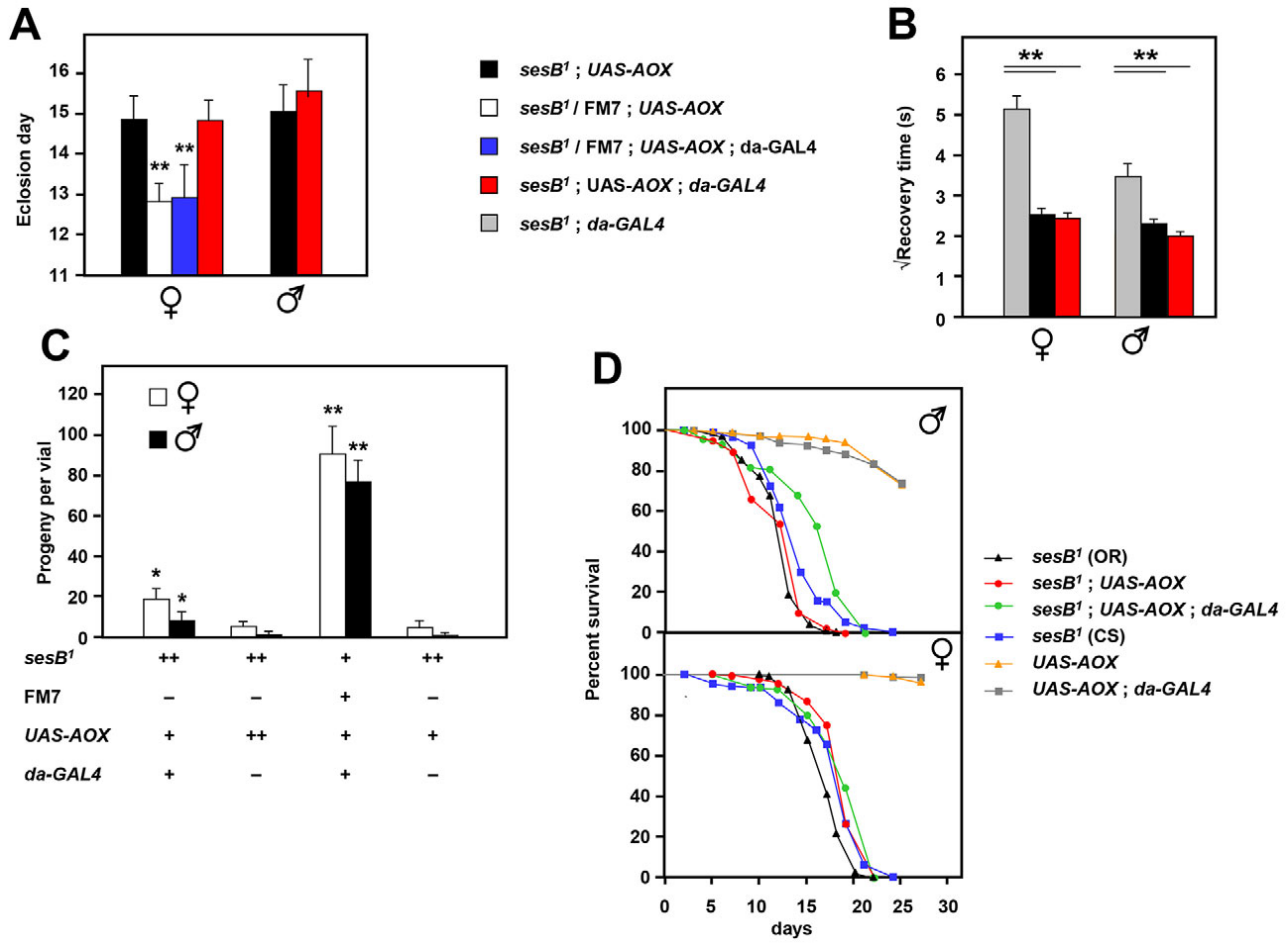
**Partial rescue of *sesB^1^* by AOX expression.** (A) The day of eclosion of flies of the genotypes and sexes indicated, reared at 22°C. Where present, *da-GAL4* and *UAS-AOX* transgenes were hemizygous. ** significant differences between *sesB^1^* and other data classes (*P*<0.01). The means±s.d. from eight to 14 replicate experiments are shown. (B) Bang sensitivity – the square-root of recovery time from mechanical shock of 3-day-old flies of the genotypes and sexes indicated, reared at 22°C; means±s.e.m. are shown. 79–215 flies of each class were analyzed individually. **P<0.01 between data classes for each sex (calculated by Student’s *t*-test with Bonferroni correction). (C) Mean numbers of offspring per vial as a result of mating females of the indicated genotypes (reared at 22°C) with wild-type (Oregon R) males. +, hemizygous; ++, homozygous; −, absence of a given marker, balancer or transgene. *P<0.05, **P<0.01 between *sesB^1^;AOX* and other data classes for each sex (Student’s *t*-test). (D) Lifespan curves for male flies of the indicated genotypes. Canton S (CS) and Oregon R (OR) are different strain backgrounds, which show curtailed lifespans similar to that of *sesB^1^*. Where present together, *da-GAL4* and *UAS-AOX* transgenes were hemizygous. When *UAS-AOX* alone was present, it was homozygous in order to control for the number of copies of the *mini-white* selectable marker. The survival curve for AOX-expressing males is significantly different from all of the control lines, including *sesB^1^* in either genetic background (log-rank test, *P*<0.001). However, given that the increase in mean lifespan was quantitatively minor, and was not seen in females, a contribution from the genetic background cannot be excluded.

**Fig. 6.**
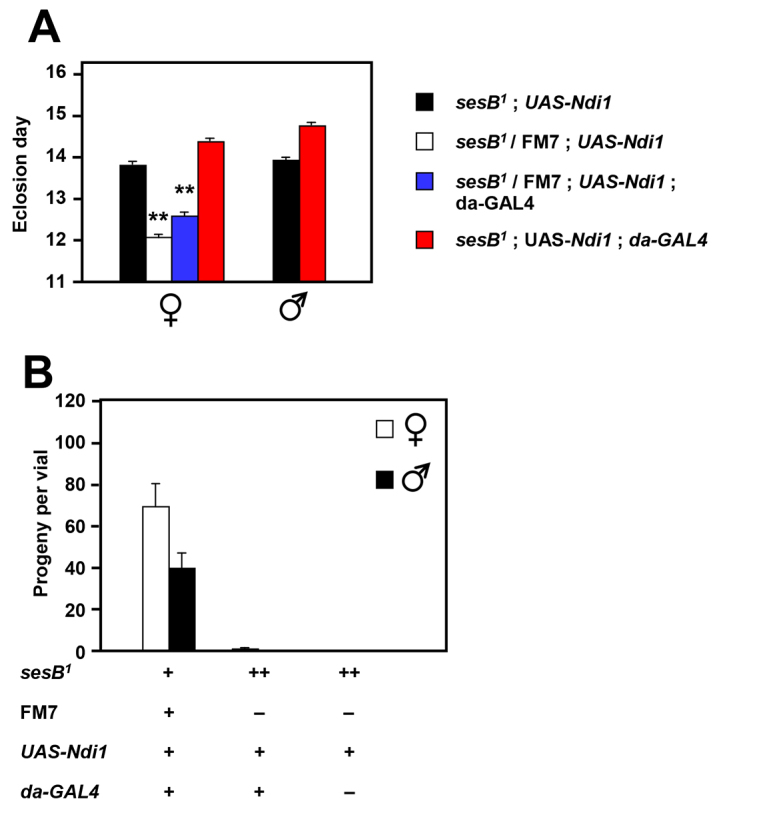
**Lack of rescue of *sesB^1^* by Ndi1 expression.** (A) The day of eclosion of flies of the genotypes and sexes indicated; means±s.e.m. from three biological replicates are shown. Where present, *da-GAL4* and *UAS-Ndi1* transgenes were hemizygous. **P<0.01 between *sesB^1^;Ndi1* and other data classes for each sex (Student’s *t*-test with Bonferroni correction where appropriate). (B) The mean numbers of offspring per vial resulting from the mating of females of the genotypes indicated with wild-type (Oregon R) males. +, hemizygous; ++, homozygous; −, absence of a given marker, balancer or transgene.

Testing showed that AOX expression was unable to rescue developmental delay (Fig. 5A), although there was, once again, a minor degree of rescue of bang sensitivity (Fig. 5B), which was not further enhanced by *da-GAL4*-driven high-level expression. Expression of the alternative mitochondrial NADH dehydrogenase Ndi1 from yeast (Fig. 6A) was also unable to rescue developmental delay and, instead, tended to increase it.

To test for the possible rescue of female sterility, we crossed *sesB^1^* females (1- to 4-day-old virgins) that also carried transgenic upstream activator sequence (*UAS*)-*AOX* (Fernández-Ayala et al., 2009), with or without the ubiquitously acting *da-GAL4* driver, with wild-type males. Control females that were heterozygous for *sesB^1^* gave substantial numbers of progeny (Fig. 5C), but, unlike non-transgenic *sesB^1^* homozygous females or the flies that had been tested previously in the BER-1 mitochondrial or *spargel* overexpressor backgrounds, those flies also endowed with *UAS-AOX* gave progeny well in excess of the ~0.1% survivor rate obtained routinely from *sesB^1^* females. In the presence of only the transgene encoding UAS-AOX, the number of progeny was ~3% of the number from control crosses, but the additional presence of the *da-GAL4* driver raised this to 15–20% (Fig. 5C). Ndi1 expression failed to rescue female sterility, with only a tiny number of progeny (Fig. 6B). Note that the experiments using the transgenes that encoded UAS-AOX and UAS-Ndi1 were performed in the same genetic background, thus the differences are biologically meaningful, as well as statistically significant.

The short lifespan of *sesB^1^* flies was marginally improved by AOX expression – AOX-expressing males showed a statistically significant increase in their mean lifespan of 3–4 days compared with that of control groups (Fig. 5D), but the effect in females was negligible (Fig. 6B). However, given that the rescue was quantitatively minor and sex-specific, a contribution of the genetic background cannot be excluded.

## DISCUSSION

In this study, we characterized the biochemical and phenotypic consequences of the *sesB^1^* mutation in the major ANT gene of *Drosophila*, and tested different strategies to complement its phenotype *in vivo*. We determined that the mutation limits respiration, causes a moderate deficiency of ATP and increases the level of lactate. Most aspects of the adult phenotype, and alterations in gene expression that affect metabolism, are shared with the *tko^25t^* mutant, revealing common underlying molecular pathways. However, *sesB^1^* flies also exhibit the distinct, and sex-specific, phenotype of female sterility, which is associated with a downregulation of a broad range of genes that are associated with oogenesis. Female sterility was overcome by the expression of AOX from *Ciona*, but this did not rescue developmental delay and only partially alleviated bang sensitivity. Crossing *sesB^1^* into the BER-1 mtDNA background produced modest apparent improvements in the developmental phenotype but had little impact on female fertility, whereas the effects of *spargel* overexpression were even more minor.

### Implications of the biochemical phenotype

The *sesB^1^* biochemical phenotype is the same as that which would be predicted if the mutation causes a decreased rate of adenine nucleotide exchange. The RCR can be construed as reflecting the degree of functional coupling between ATP synthesis and respiratory electron flow. A low RCR (Fig. 1A), combined with ATP deficiency (Fergestad et al., 2006; Terhzaz et al., 2010), as verified here (Fig. 1B), thus reflects either increased proton leak across the inner mitochondrial membrane and/or a limitation on ATP synthesis, either of which are plausible molecular consequences of the *sesB^1^* mutation, which affects the final transmembrane segment of the protein. Despite modeling in yeast, which supported uncoupling as the mechanism of adPEO mutations in ANT1 (Wang et al., 2008), we favor the latter explanation because it is more easily reconciled with the fact that the mutation is recessive and is parsimonious with the reported biochemical phenotype of the V298M adPEO mutation (Kawamata et al., 2011), which affects the residue adjacent to that mutated in *sesB^1^*. Previously, it was shown that decreased expression of wild-type *sesB* decreased the net proton conductance of the inner mitochondrial membrane, whereas its overexpression increased it (Brand et al., 2005). Thus, increased proton leak seems an unlikely explanation for the effect of the *sesB^1^* mutation. Furthermore, the apparent decrease in the amount of functional cytochrome oxidase (Fig. 1D) is a plausible response to a limitation of ATP synthesis as a mechanism to restrict electron flow and prevent increased membrane potential that would result in overproduction of ROS, as seen in the mouse *Ant1* knockout (Esposito et al., 1999). Increased proton leak would be expected to produce, if anything, an opposite response to that observed here.

Based on the findings from transcriptomics and metabolite analysis, we found evidence of a remodeling of metabolism that should favor glycolytic ATP generation – pyruvate was converted to lactate to regenerate NAD^+^, fatty acid and amino acid mobilization and catabolism were induced, as well as the induction of anaplerotic pathways to compensate for a presumed decrease in the mitochondrial import of pyruvate. Furthermore, the major stress-response gene, *Hsp22*, a mitochondrial chaperone of the lens-crystallin superfamily, was upregulated (Morrow et al., 2000; Michaud et al., 2002). As a programmed response to transient mitochondrial insufficiency, we hypothesize that this is sufficient to sustain metabolism and support development, even if the outcome is sub-optimal, yielding flies that are bang-sensitive and defective in reproduction.

### Phenotypic overlap with *tko^25t^*

The *sesB^1^* phenotype has many similarities with that of *tko^25t^*, including decreased ATP levels, developmental delay, bang sensitivity, male courtship defect, hearing impairment and a similar remodeling of the metabolic component of gene expression (Toivonen et al., 2001; Fernández-Ayala et al., 2010; Chen et al., 2012). When considering potential future treatments of these distinct classes of mitochondrial disease, such common molecular pathways might prove fruitful targets.

One major difference between the two mutants is that *tko^25t^* has an essentially normal lifespan (Chen et al., 2012), whereas that of *sesB^1^* is drastically shortened (Fig. 1D). The adult fly is essentially post-mitotic; therefore, in contrast with development – when active cell growth and cell division take place – mitochondrial protein synthesis in adults is required only to keep pace with protein and organelle turnover. A greatly decreased capacity for mitochondrial protein synthesis, as in *tko^25t^*, might thus have severe effects on development, yet still be above the rate that is required for turnover in the adult. If this is the case, the capacity for OXPHOS should gradually increase in *tko^25t^* adults, eventually reaching a level similar to that of wild-type flies, with only a minimal, and probably undetectable, effect on aging.

By contrast, the limitation on respiration and OXPHOS that is conferred by decreased ANT activity in *sesB^1^* should persist in the adult, imposing chronic stress and probably accounting for the drastically curtailed lifespan. One obvious potential stressor that is already associated both with ANT dysfunction and lifespan regulation is excess ROS production. AOX decreases mitochondrial ROS production, even under non-inhibited conditions (Fernández-Ayala et al., 2009; Sanz et al., 2010b), although this is not understood mechanistically. The modest lifespan extension that is produced by AOX, at least in *sesB^1^* males, could thus be mediated through an effect on ROS. However, this increase is quantitatively too small to exclude an effect of genetic background.

A second major difference between *sesB^1^* and *tko^25t^* is that *tko^25t^* females are fertile, despite a reduced rate of egg laying (Fernández-Ayala et al., 2010), whereas *sesB^1^* females are sterile, giving rise to eggs that can be fertilized but fail to complete embryogenesis. This is also reflected in decreased ovary size (Fig. 1G) and the downregulation of many genes that are connected with oogenesis in *sesB^1^* females (Table 3; supplementary material Table S5). Although the number of survivors was slightly increased in the BER-1 mtDNA background or upon increased *spargel* expression, only the expression of AOX, driven by *da-GAL4*, was able to produce a pronounced rescue. By contrast, expression of Ndi1, which bypasses (and also alleviates ROS production at) complex I (Sanz et al., 2010a), had only a negligible effect on female sterility (and lifespan), excluding background effects from the *da-GAL4* driver or the *w^1118^* recipient that was used to generate the transgenic lines. AOX was partially effective at alleviating female sterility, even when expressed only at a low level (without the *da-GAL4* driver), which was also the case for its partial rescue of bang sensitivity, reminiscent of previous observations on its rescue of the locomotor defect that is caused by deficiency of *dj-1β* (Fernández-Ayala et al., 2009).

The fact that AOX partially rescued fertility, whereas Ndi1 did not, can be interpreted in several alternative ways. The first possibility is that rescue by AOX involves the mobilization of substrates that supply electrons downstream of complex I (such as succinate or glycerol-3-phosphate). AOX can transfer these electrons to oxygen without net proton pumping, circumventing the limitation on electron flow that is imposed by the coupling mechanism caused by ANT insufficiency. The second is that the observed downregulation of complex IV in *sesB^1^* flies limits any facilitation of electron flow by Ndi1. AOX bypasses complex IV and so should be unaffected. The third possibility invokes a different metabolic effect of AOX, such as decreased ROS production.

Because the standard UAS-GAL4 system is inactive in the female germline (Rørth, 1998), the AOX-mediated rescue of sterility must be attributable to its expression in somatic tissues, or in the embryo itself. Because *sesB^1^* females are sterile when mated to wild-type males, it is evident that zygotic expression of even *sesB* itself cannot rescue sterility. Thus, it seems unlikely that AOX could do so. Instead, we favor the idea that insufficiency of mitochondrial adenine nucleotide transport in one or more somatic cell types in the ovary leads to a dysregulation of oogenesis that can be rescued by AOX. The fact that so many ovary-specific genes are downregulated in *sesB^1^* is consistent with a general failure of oogenesis rather than a specific metabolic abnormality in developing oocytes.

The somatic cells of the ovary, in particular the follicle cells, play key roles in the patterning of the developing oocyte (Deng and Bownes, 1998; Poulton and Deng, 2007), and many molecular signaling pathways are involved (Roth et al., 1995; Neuman-Silberberg and Schüpbach, 1996; Rørth et al., 2000; Yamamoto et al., 2007; Stein et al., 2013; Fregoso Lomas et al., 2013), some of which are functionally conserved in vertebrates (Charlier et al., 2012). Moreover, cell proliferation and migration – crucial to the developmental function of the follicular epithelium – are commonly regulated by metabolite or redox signaling (Veal and Day, 2011; Hurd et al., 2012). ANT dysfunction might interfere with this signaling, whereas AOX might restore it.

### Modifiers of the *sesB^1^* phenotype

ANT is an abundant component of the protein-rich inner mitochondrial membrane, probably reflecting a quantitative requirement for a high rate of adenine nucleotide transport to match the capacity of ATP synthase. For this reason, a decrease in ANT activity is likely to become limiting for ATP synthesis, with a small margin for compensation by increasing the expression or stabilization of the protein. However, globally increased mitochondrial biogenesis mediated by, for example, *PGC-1α* (*spargel*) would be a plausible compensation strategy. In previous studies, this pathway has been implicated in the partial rescue of *tko^25t^* by the BER-1 mtDNA background (Chen et al., 2012). However, the modest phenotypic improvement in *sesB^1^* that was brought about by BER-1 mtDNA was not accompanied by increased *spargel* expression. Moreover, a twofold overexpression of *spargel* had no effect on the developmental delay of *sesB^1^* flies, indicating that a different mechanism underlies the effects of BER-1-mediated rescue.

Compared with control strains, BER-1 mtDNA contains a number of sequence polymorphisms, which affect both protein- and RNA-encoding genes (Chen et al., 2012). These might act in a variety of ways to modify the *sesB^1^* phenotype – e.g. by altering the functional properties of the OXPHOS system, the architecture of the inner mitochondrial membrane or the output of mitochondrial protein synthesis. An effect on mitochondrial biogenesis that is independent of the *spargel* pathway can also not be excluded. In order to understand how BER-1 influences *sesB^1^* it will be useful to compare in detail the properties of isolated mitochondria from *sesB^1^* and control flies containing BER-1 versus control-strain mtDNA. Regardless of the precise mechanism, our findings provide support for the idea that the mitochondrial genotype can act as a modifier in humans with recessive *ANT1*-linked disease (Strauss et al., 2013).

### *sesB^1^* as a disease model and implications for therapy

Despite its ability to rescue female sterility, AOX expression produced no improvement in the developmental delay of *sesB^1^* flies, consistent with the notion that this phenotype is, most probably, attributable to the limitation on ATP production that is imposed by *sesB^1^*. ATP deficiency, specifically at synapses, has also been suggested to underlie the phenotype of a second mutant allele of *sesB*, *sesB^org^*, which confers temperature-sensitive paralysis (Rikhy et al., 2003).

Overall, our findings indicate that there are at least two pathways by which ANT deficiency can lead to pathological tissue dysfunction, and the *sesB^1^* model now allows these to be explored in further detail. The first pathway is through bioenergetic insufficiency, manifesting as an ATP deficit, of which developmental delay is the principal indication. Its apparent alleviation by the BER-1 mtDNA background might have substantial implications for therapy, if the mechanism can be elucidated. Conversely, despite a large interest in therapeutically boosting PGC1-α expression (Wenz et al., 2008; Srivastava et al., 2009; Dillon et al., 2012; Chaturvedi and Beal, 2013; Komen and Thorburn, 2014; Noe et al., 2013; Hofer et al., 2014), our results indicate that modulating PGC1-α activity might not be a viable strategy for alleviating the features of ANT1 diseases that are associated with bioenergy deficit. Nevertheless, much stronger activation of the pathway, such as using *UAS-spargel*, might yet be worth testing.

The second pathway by which ANT deficiency produces a pathological outcome is the one alleviated by AOX, involving disturbed metabolic or redox homeostasis.

Ubiquitous AOX expression in both the fly and the mouse is essentially benign (Fernández-Ayala et al., 2009; El-Khoury et al., 2013). The benefit of this in future therapy (El-Khoury et al., 2014) is already indicated by the ability of AOX to alleviate the pathological defects that are caused by functional loss of complex IV (Fernández-Ayala et al., 2009; Kemppainen et al., 2014) or other mitochondrial components that are needed for OXPHOS, such as DNA polymerase γ (Humphrey et al., 2012). Here, we extend this range of targets to include ANT, but with an important caveat. Clearly, only some of the phenotypes that are associated with pathological ANT dysfunction can be mitigated by AOX. Thus, any potential use of AOX in therapy for ANT-related, and other mitochondrial, disorders should be based on a clear understanding of the underlying pathological mechanism and the metabolic status. These might differ systematically between tissues, according to nutritional state or under influences of the genetic background.

## MATERIALS AND METHODS

### Flies, maintenance and behavioral assays

*Drosophila melanogaster* lines were as described previously (Toivonen et al., 2001; Fernández-Ayala et al., 2009; Sanz et al., 2010a; Kemppainen et al., 2009; Chen et al., 2012) or from stock centers (*sesB^1^*, *bss*, *sda*, *Mef2-GAL4*, *mito-HA-GFP*). The *srl^GR^* line (Tiefenböck et al., 2010), containing an additional genomic copy of the *spargel* gene, was a kind gift from Christian Frei (ETH Zürich, Switzerland). Flies were maintained at 25°C on standard medium with supplements, as described previously (Fernández-Ayala et al., 2009) except where indicated. Time to eclosion, bang sensitivity (at 25°C), response to sound and male courtship were measured as described previously (Toivonen et al., 2001). Lifespan at 25°C was determined as described previously (Sanz et al., 2010a).

### Microscopy

The dissected ovaries from 2-day-old virgin females were placed on a slide in a droplet of PBS, and images were captured with and without coverslips by using a Nikon SMZ745T microscope with DS-Vi1 camera. The embryos were collected from mating chambers, dechorionated, placed on two-sided tape that was attached the bottom of a Petri dish and then minimally overlaid with dissection medium (137 mM NaCl, 5.4 mM KCl, 0.17 mM NaH_2_PO_4_, 0.22 mM KH_2_PO_4_, 33.3 mM glucose, 43.8 mM sucrose and 9.9 mM HEPES, pH 7.4). Embryos were imaged as described above. Indirect flight muscle mitochondria of flies that expressed GFP that was targeted to the mitochondria were imaged by using a Nikon Eclipse-Ti microscope (Nikon Instruments, Melville, NY) plus an Andor Neo sCMOS camera (Oxford Instruments, Abingdon, UK) with a 40× objective. The flies were grown for 2 days, after which the thoraxes were fixed with phosphate buffered 10% formalin pH 7.0 (FF-Chemicals, Haukipudas, Finland) for 30 minutes, washed with PBS three times for 10 minutes, after which further dissection of muscle fibers was performed with a 27-gauge needle attached to a 1 ml syringe.

### Metabolite analysis

ATP levels were assayed using the ATP Determination Kit (Life Technologies, Carlsbad, CA) according to the manufacturer’s instructions. For each sample, a set of ten flies were homogenized in 100 μl of 6 M guanidine hydrochloride and then centrifuged at 12,000 *g*_max_ for 5 minutes at 4°C. Supernatants were diluted tenfold in TE buffer (10 mM Tris-HCl, 1 mM EDTA, pH 8.0), and 10 μl of the diluted sample was mixed with the reaction solution. Different genotypes were processed in biological triplicates and luminescence was measured using a Chameleon plate reader (Hidex, Turku, Finland).

Lactate was assayed by using a BioVision Lactate Colorimetric/Fluorometric Assay Kit (BioVision, Milpitas, CA) according to the manufacturer’s instructions. Samples of 30 flies per genotype were homogenized in 300 μl of lactate assay buffer and centrifuged at 12,000 *g*_max_ for 5 minutes at 4°C. Each genotype was analyzed in triplicate and sample fluorescence was recorded using the Hidex Chameleon plate reader.

### Measurements of mitochondrial function

Mitochondria were isolated for oxygraphic analysis and blue native electrophoresis from ~200 flies per genotype, as previously described (Miwa et al., 2003). Protein concentrations were determined by the Bradford method (Bradford, 1976). Oxygen consumption of the isolated mitochondria was measured in a Clark-type electrode chamber (Hansatech Oxyterm system) in the presence of different substrate mixes (5 mM sodium pyruvate plus 5 mM proline for complex-I-linked respiration, 20 mM sn-glycerol 3-phosphate for complex-III-linked respiration) before and after the addition of 1 mM ADP, as described previously (Fernández-Ayala et al., 2009). The oxygen consumption in the presence of the different substrate mixes was then used to compute the RCR. Blue native electrophoresis and histochemical staining for complex I and IV activity were performed on mitochondrial extracts, as described previously (Sanz et al., 2010a).

### RNA isolation and analysis

RNA extraction, microarray analysis using the GeneChip^®^ Affymetrix *Drosophila* 2.0 array (Affymetrix, Santa Clara, CA) and quantitative RT-PCR were performed as described previously (Fernández-Ayala et al., 2009; Fernández-Ayala et al., 2010). RNA isolations were performed in triplicate from batches of 30 2-day-old males and virgin females. Raw microarray data was normalized and analyzed using Genespring GX software (Agilent Technologies, Santa Clara, CA). Previous raw data from *tko^25t^* mutant flies (Fernández-Ayala et al., 2010) was incorporated for comparative analysis, for which data normalization and analysis was performed afresh, grouping all values for the control samples. Genes that showed altered expression were clustered according to GO classes, using GO Term Mapper (Lewis-Sigler Institute for Integrative Genomics, Princeton University, NJ). For quantitative RT-PCR, cDNA was synthesized using the SuperScript^®^ kit (Life Technologies, Carlsbad, CA) or, for *spargel* RNA, using a High Capacity cDNA Reverse Transcription kit (Applied Biosystems). Analysis used a StepOnePlus instrument (Life Technologies, Carlsbad, CA) with the manufacturer’s SYBR^®^ Green PCR reagents and customized primers, as listed in supplementary material Table S13. Normalization to *RpL32* RNA was performed as described previously (Fernández-Ayala et al., 2009).

### DNA extraction and sequencing

For sequencing, genomic DNA was prepared from batches of ten to 30 adult flies, as described previously (Fernández-Ayala et al., 2009). A 500 bp fragment of the *sesB* gene was amplified using primer pair sesB1F: 5′-GAGGAAGGAAGGGTTCTTGC-3′ and sesB1R: 5′-CATCGTGTCCTATCCCTTCG-3′. The purified PCR products were sequenced using the same primers (Life Technologies, 3130x1 Genetic Analyzer).

### Statistical analysis

In all experiments, the means were compared, as appropriate, by Student’s *t*-test or ANOVA, with Bonferroni correction for multiple comparisons.

## Supplementary Material

Supplementary Material
