# Supplementary material for: Phenotypic rescue of a Drosophila model of mitochondrial ANT1 disease
Source: Dis Model Mech. 2014 May 8;7(6):635–48. doi: 10.1242/dmm.016527 (PMC4036471; doi:10.1242/dmm.016527)
Supplement: Supplementary Material [file supp_7_6_635__index.html]

Phenotypic rescue of a Drosophila model of mitochondrial ANT1 disease — Supplementary Material 

# Phenotypic rescue of a *Drosophila* model of mitochondrial ANT1 disease

## DMM016527 Supplementary Material

**Files in this Data Supplement:**

- **Supplementary Material**
