# Supplementary material for: Phenotypic rescue of a Drosophila model of mitochondrial ANT1 disease
Source: Dis Model Mech. 2014 May 8;7(6):635–48. doi: 10.1242/dmm.016527 (PMC4036471; doi:10.1242/dmm.016527)
Supplement: Supplementary Material [file supp_7.6.635_DMM016527.pdf]

Phenotypic rescue of a Drosophila model of mitochondrial ANT1 disease

Suvi Vartiainen, Shanjun Chen, Jack George, Tea Tuomela, Kaisa R. Luoto, Kevin M.C. O'Dell & Howard T. Jacobs

SUPPLEMENTARY MATERIAL

SUPPLEMENTARY FIGURE LEGENDS

Figure S1

BNE gel images

Full images of the BNE gel (Fig. 2D) histochemically stained for complex IV activity (left) and showing only the Serva G protein stain prior to processing for histochemistry (right). The right hand gel image shows also the migration of molecular weight markers used to extrapolate the apparent sizes of the complexes arrowed in the main figure.

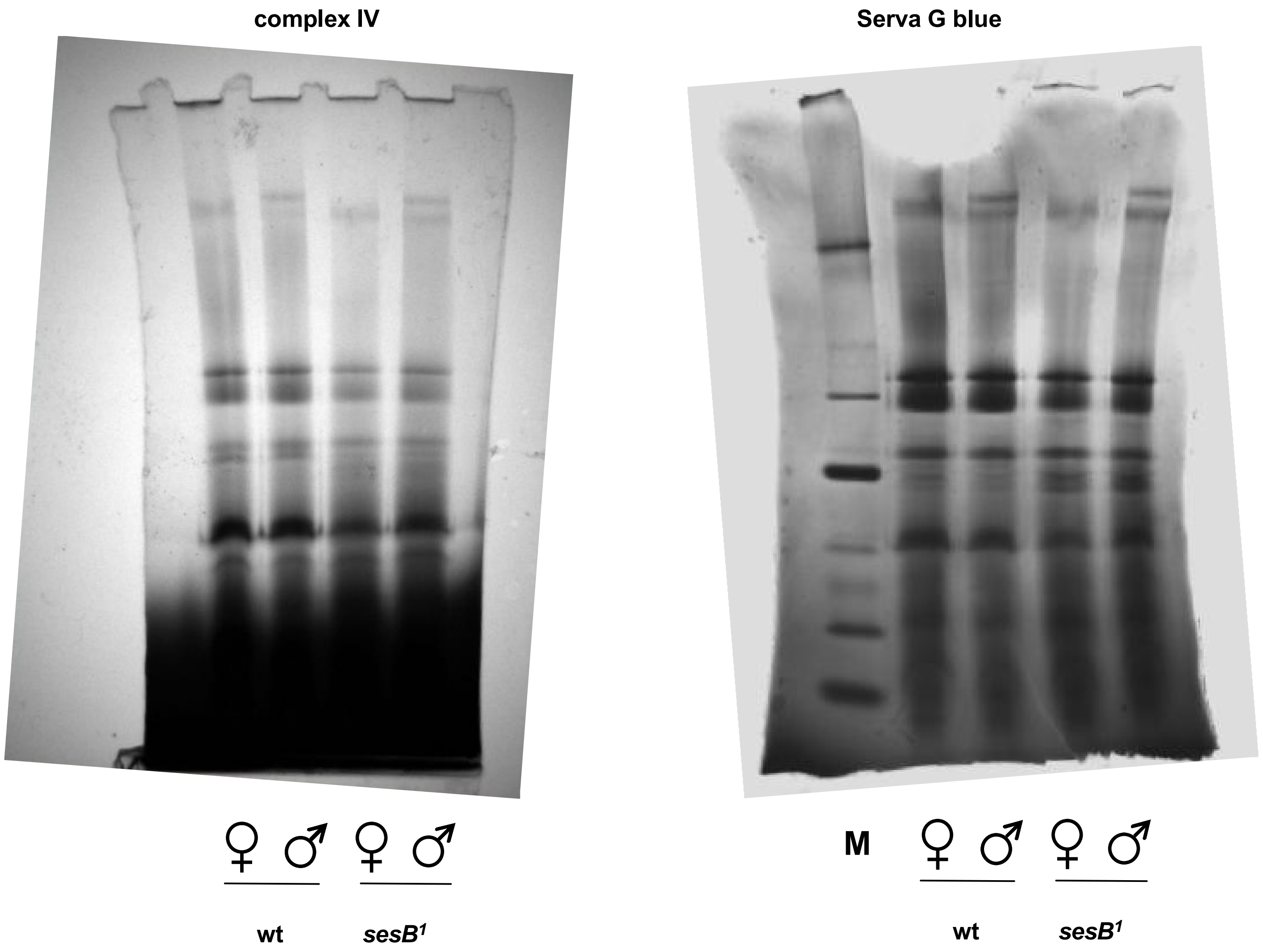

Figure S1, Vartiainen et al

SUPPLEMENTARY TABLES

Tables S1-S12 are displayed in the accompanying Excel file (Tables S1-S12.xls).

Download Tables S1-S12

Table S13

Primers used for QRTPCR (all shown 5′ to 3′)

**Symbol**	**Forward primer**	**Reverse primer**
bcd	ACGAGCCATTAACACCCAAG	GCTCTTGTCCAGACCCTTCA
Cys	GGAAGGAGGCTCTGGAACTT	CTGCTTTTTGTCGGATCCAT
Fbp1	GTCCTGCTCGACAAGGATGT	GGTTCCAGGCCATAGACAGA
fbp	GCGAGGAGGTCAAGAAACTG	AGATTGAACCGATCGACACC
Hex-C	TACATCTGCGAGTGCGTAGC	GCTTTCACCAGCTTTTGGAG
Hsp22	TGGCTATAGCTCCAGGCACT	AGTCTGCTCGATGGTCACCT
Lip3	CAGGCCACTTCTCCTTATGC	GGATTACGGAGAGCGGTGTA
Obp99b	AAGGTTCTCATCGTTCTCCTATTG	GGGTACTGCCACTTCTTGTACTTC
Pepck	CAGTGCTTCGAGGACTCTCC	CTCTCTATGGCAGCCACCTC
CG8093	AAGTCTGGGCTTGGCTAATG	GGATGTAACCATCCGAGGTC
srl	CCTCGACTACATTCGGTGCT	AGACGTGCCTTCTGTCGTTC
RpL32	AGGCCCAAGATCGTGAAGAA	TGTGCACCAGGAACTTCTTGAA
